# Advance Research on the Pre-Harvest Sprouting Trait in Vegetable Crop Seeds

**DOI:** 10.3390/ijms242417171

**Published:** 2023-12-06

**Authors:** Yixin Qu, Yaqi Zhang, Zhongren Zhang, Shanshan Fan, Yu Qi, Fang Wang, Mingqi Wang, Min Feng, Xingwang Liu, Huazhong Ren

**Affiliations:** 1 Department of Vegetable Science, College of Horticulture, China Agricultural University, Beijing 100193, China; 2 Sanya Institute, China Agricultural University, Sanya 572019, China

**Keywords:** pre-harvest sprouting, vegetable crops, plant hormones, abscisic acid

## Abstract

Pre-harvest sprouting (PHS), the germination of seeds on the plant prior to harvest, poses significant challenges to agriculture. It not only reduces seed and grain yield, but also impairs the commodity quality of the fruit, ultimately affecting the success of the subsequent crop cycle. A deeper understanding of PHS is essential for guiding future breeding strategies, mitigating its impact on seed production rates and the commercial quality of fruits. PHS is a complex phenomenon influenced by genetic, physiological, and environmental factors. Many of these factors exert their influence on PHS through the intricate regulation of plant hormones responsible for seed germination. While numerous genes related to PHS have been identified in food crops, the study of PHS in vegetable crops is still in its early stages. This review delves into the regulatory elements, functional genes, and recent research developments related to PHS in vegetable crops. Meanwhile, this paper presents a novel understanding of PHS, aiming to serve as a reference for the study of this trait in vegetable crops.

## 1. Introduction

**Figure 1:**
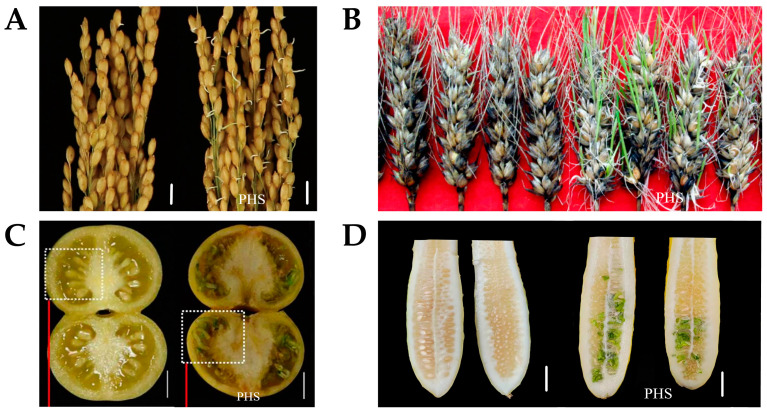
Morphology of pre-harvest sprouting in plants [4,5,6]. (**A**) Pre-harvest sprouting in rice [4]. (**B**) Pre-harvest sprouting in wheat [5]. (**C**) Pre-harvest sprouting in tomato [6]. (**D**) Pre-harvest sprouting in cucumber.

Pre-harvest sprouting (PHS) is a botanical occurrence during which seeds germinate on the plant before harvest without, or after, a short dormancy (Figure 1). In the context of food crops, PHS is frequently referred to as “viviparous,” due to the unique arrangement of seeds (Figure 1A,B). In horticultural crops, especially those like melons where seeds are enclosed within the fruit, it is termed “seed germination in melon” (Figure 1C,D) [1]. The implications of PHS vary depending on whether the crop primarily treats seeds as a marketable product or if the focus is on the maturity of the crop itself. In the case of food crops, where seeds are the primary commodity, PHS significantly impacts both the quantity of seeds produced and the overall yield [2]. However, when considering vegetable crops that are categorized as commercially and physiologically mature, the effects of PHS are different. In commercially mature vegetable crops like cucumbers, PHS primarily affects the seed production rate, rather than jeopardizing the commercial viability of the crops. On the other hand, in physiologically mature vegetable crops like tomatoes, PHS has more profound consequences. It may lead to a reduction in carbohydrates, soluble solids, vitamin C, and other components in the fruit, ultimately affecting its flavor and overall marketability [3].

Vegetables are rich in essential nutrients that contribute to maintaining human health and fulfilling our nutritional requirements. In China, there is a large demand for vegetable seeds, due to the widespread cultivation of vegetables. To increase income in modern agriculture, it is imperative to continually boost yield per unit and the overall product quality, with the pivotal factor being the quality of the seeds. Vegetable crops often face the challenge of PHS, which can significantly influence the economic viability and the quantity of high-quality vegetable seeds produced. This, in turn, exerts an impact on the cultivated area and the ultimate crop yield [7].

Previous research has primarily focused on PHS in plants like Arabidopsis, rice, wheat, and maize. Many significant genes, such as *TaMFT*, *TaPHS1*, *AtABI3*, *AtDOG1*, *AtLEC 2*, *ZmVP1-10*, *OsSdr4*, *OsDSG1*, *OsABI3*, *OsABI5*, *OsPHS8*, *OsPHS9*, *OsNCED3*, *OsVP1*, *OsPDS*, *OsLCU*, *OsFbx352*, *OsMFT2*, *OsZDS*, and *OsCRTISO* have been identified. These genes have been linked to various aspects of PHS, including their roles in the biosynthesis, catabolism, perception, and signaling pathway of abscisic acid (ABA) and gibberellins (GA). Similar to germination, PHS also involves intricate physiological processes and a diverse array of compounds. However, the precise regulatory mechanisms governing these processes remain largely mysterious, especially in the context of vegetable crops. At present, research on vegetable crops focuses primarily on gene discovery, physiology, and metabolism. However, only a few resistant genes have been identified, and there is a lack of corresponding molecular regulatory mechanisms or comprehensive functional analyses of specific genes, thus limiting the scope of current research. In this review, we aim to provide a comprehensive summary of recent literature that delves into the genetic regulation and molecular mechanisms of PHS, thereby contributing to a deeper understanding of PHS in vegetable crops and offering valuable insights for further research in this important field.

## 2. Pre-Harvest Sprouting Tightly Regulated by Plant Hormones

PHS is a peculiar occurrence in seed germination, closely linked to the plant hormones ABA and GA. These phytohormones are well-established regulators of seed germination, especially in food crops. While the roles of ABA and GA in PHS are more thoroughly understood in food crops, their involvement has also been verified in vegetable crops.

In cucumber, transcriptome analysis comparing materials prone to PHS and those resistant to it reveals that the differentially expressed genes (DEGs) are significantly enriched in plant hormone signal transduction pathways [8]. The majority of the DEGs are associated with ethylene and ABA, emphasizing their pivotal role in controlling cucumber PHS. Interestingly, applying exogenous ABA to the surface of cucumber fruit completely prevents PHS. Meanwhile, there is no discernible difference in germination rates between the seeds harvested from ABA-treated and untreated cucumber fruits [9]. This suggests that exogenously sprayed ABA may affect cucumber germination primarily through the fruit tissues, rather than through the seeds themselves.

Additionally, analyzing ABA and GA levels in seeds and capsule coats in varieties of cucumber prone to PHS and resistant to PHS, it is evident that the ABA content and ABA:GA3 ratio in seeds of prone-to-PHS varieties are lower than those in resistant-to-PHS seeds [10]. Zucchini and rape, two other examples, exhibit similar patterns; prone-to-PHS varieties have a higher GA content and a lower ABA content, resulting in a lower ABA:GA ratio, which leads to early seed germination [11,12]. In the case of tomatoes, research has shown that ABA plays a critical role in seed germination. A mutant lacking ABA germinates faster and at a greater germination rate compared with a mutant lacking GA. ABA-deficient mutants exhibit significantly lower levels of ABA in the mesoderm and endosperm compared to wild type tomatoes, directly resulting in PHS during seed development [13]. In the fruit ripening mutant *rin*, the degree of germination varies as tomato seeds are exposed to various ABA concentrations, with prone-to-PHS varieties being more susceptible to higher ABA levels [14].

It is important to note that the variation in seed dormancy or PHS rate is not necessarily linked to the absolute ABA content but rather to the sensitivity of the embryo to ABA [15]. The precise connection between seed germination variations and embryo sensitivity to ABA remains unknown in vegetable crops. This is partly due to the fact that the technique for determining embryo sensitivity to ABA in crops like rice has not yet been widely employed in vegetable crops. Nonetheless, these findings strongly suggest that endogenous plant hormones like ABA play a crucial role in regulating this complex germination event.

## 3. Pre-Harvest Sprouting as a Quantitative Trait Affected by Various Factors

In food crops like rice and wheat, PHS has been consistently established as a quantitative trait, with the identification of numerous quantitative trait loci (QTLs) distributed across different chromosomes. In vegetable crops, some relevant QTLs are also identified (Table 1). For instance, in pea beans, PHS has been discovered to be a polygenic quantitative trait with a clear maternal impact [16]. In Chinese cabbage, a cross between prone-to-PHS and resistant-to-PHS varieties revealed that the progeny exhibited a dominant trait of resistance to PHS [17]. These findings indicate that the PHS is regulated by both cytoplasmic and nuclear genes. Subsequent research identified a candidate QTL associated with PHS located within a 796 kb region on chromosome 3. In this QTL, two candidate genes, *Bra013201* and *Bra012635,* were chosen, and their Arabidopsis homologs are involved in the ABA biosynthesis pathway [18]. Interestingly, their subcellular localization revealed that these two genes were expressed in the nucleus and cytoplasm, in agreement with prior investigations. In oilseed rape, Feng identified 5 QTLs associated with PHS, namely *qPHS-2-a*, *qPHS-2-b*, *qPHS-2-c*, *qPHS-2-d*, and *qPHS-7* [12]. In cucumber, the inheritance of PHS is explained by one major gene with additive-dominance effects and additive-dominance polygene (D-1 model) [19], and a 0.53 Mb QTL was found on chromosome four [20]. Two candidate genes, *Csa4G622760* and *Csa4G622800*, annotated as chalcone-like isomerase and methionine sulfoxide reductase, were chosen, based on gene annotation and fluorescence quantitative analysis. Further analysis indicated these two genes may be related to flavonoids, which have been confirmed to be involved in the regulation of PHS resistance in plants like Arabidopsis [21].

Collectively, the genetic nature of PHS is influenced by a multitude of genetic, physiological, and environmental factors which affect PHS primarily by modulating the content and interactions of plant hormones. The interplay between these factors results in the unique and often complex characteristics of PHS in different plant species and varieties. Understanding how these factors interact is crucial for comprehensively addressing and managing PHS in agriculture and crop production.

### 3.1. Plant Hormone-Related Genes Play a Significant Role in the Pre-Harvest Sprouting Trait

**Figure 2:**
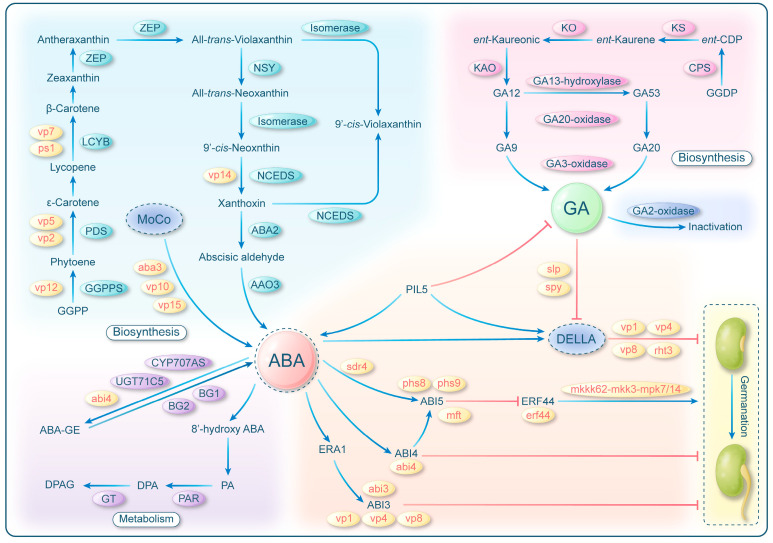
Regulatory networks of ABA and GA in seed germination. Plant hormones, such as (ABA) and gibberellin (GA), play pivotal roles in the regulation of seed dormancy and germination. The balance between germination and dormancy is finely tuned by modulating the ABA:GA ratio. Mutation in key enzymes, such as GGPPS, PDS, LCYB, and NCEDS in the ABA biosynthesis pathway, results in reduced ABA content, consequently decreasing the ABA:GA ratio and leading to PHS. The inhibition of MoCo biosynthesis, a crucial coenzyme factor for ABA biosynthesis, or its binding to ABA, also diminishes ABA content, contributing to PHS. Additionally, enhancing ABA metabolism reduces the ABA:GA ratio, further influencing seed germination. Transcription factors ABI3, ABI4, and ABI5 downstream of ABA signaling also affect PHS. ABI5, a core element of the ABA signal transduction pathway, induces seed dormancy by inhibiting the promoting effect of ERF44 on seed germination. Suppression of ERF44 and its downstream signals can also induce PHS. In the GA pathway, mutations in key enzymes in synthetic and metabolic pathways can alter GA content, subsequently affecting the ABA:GA ratio and leading to PHS. GA promotes germination by inhibiting the inhibitory effect of DELLA on seed germination. Mutations in key enzymes or transcription factors in this pathway can also indirectly impact ABA:GA ratio, thereby causing PHS. Key mutants are highlighted in red at their respective locations.

In wheat and rice, several genes responsible for PHS have been identified and characterized, with their functions mostly involved in the biosynthesis, metabolism, and signaling pathway of ABA and GA (Figure 2). One key pathway that results in PHS involves interference with ABA biosynthesis. Mutations in genes like *Viviparous 2* (*VP2*) [22], *Viviparous 5* (*VP5*) [22], *Pink Scutellum 1*/*Viviparous 7* (*PS1*/*VP7*) [23], and *Viviparous 12* (*VP12*) [24] hinder the production of ABA biosynthetic precursor carotenoids, impair ABA biosynthesis, and disrupt chloroplast development. As a result, in such mutants, the absence of functional chloroplasts leads to their eventual demise, since they cannot fix carbon through photosynthesis. For instance, *VP5* encodes phytoene desaturase that inhibits the conversion of phytoene to ε-carotene, thereby blocking the formation of ABA biosynthetic precursor carotenoids. The final steps of the ABA biosynthesis pathway can also be regulated by genes like *ABA Deficient 3* (*ABA3*) [25,26], *Viviparous 10* (*VP10*) [27,28], *Viviparous 14* (*VP14*) [29], and *Viviparous 15* (*VP15*) [30]. These genes regulated ABA content without altering carotenoid biosynthesis, allowing the plant to survive and leading to PHS. For instance, the *VP10* mutation disrupts the production of the cofactor for ABA synthesis, MoCo, thus leading to reduced ABA production. Such mutations in these genes result in a significant reduction in ABA content, a characteristic feature of ABA-deficient mutants.

PHS is regulated not only by the ABA production but also by ABA signaling. One of the most extensively researched genes related to PHS in plants is *Viviparous1* (*VP1*), the homolog of *ABI3* in rice and maize. *VP1*, a transcription factor sensing ABA in maize, plays a key role in amplifying the hormonal responses unique to seeds [31]. Furthermore, *ZmVP1* has been shown to inhibit α-starch hydrolysis and specifically disrupt GA signaling, thereby inhibiting germination [32]. Moreover, *VP4* [33] and *VP8* [34] are altered in a different way in genes than *VP1*, but both have an impact on the transcription of *VP1* and, consequently, ABA signaling. PHS is also regulated by several other genes involved in ABA signaling and the sensitivity of the embryo to ABA is also controlled by other genes, including *Ethylene-responsive Factor 44* (*ERF44*) [35], *Seed Dormancy 4* (*Sdr4*) [36,37], *Pre-harvest Sprouting 8* (*PHS8*) [38], *Pre-harvest Sprouting 9* (*PHS9*) [39], *Abscisic Acid Insensitive 3* (*ABI3*) [38], *Abscisic Acid Insensitive 5* (*ABI5*) [35,38], *Mother of Ft and Tel* (*MFT*) [40], and *MKKK62-MKK3-MPK7/14* [31]. The ABA content does not significantly differ in mutants of most of these genes. Instead, what characterizes ABA desensitization mutants is their inability to transmit the ABA signal downstream [41].

Another significant hormone that controls seed germination is GA. Through its negative regulation of DELLA, GA controls various physiological processes, including seed germination and plant growth. In GA-deficient mutants, seed dormancy is reduced, leading to the development of PHS [42].

Furthermore, there is a complex crosstalk between ABA and GA, which extends beyond their antagonistic interaction during seed dormancy and germination. Several genes, such as *Abscisic Acid Insensitive 4* (*ABI4*) [43], *SLEEPY1 (SLP)* [44], and *SPINDLY* (*SPY*) [45], work in concert to control the production or breakdown of ABA and GA, influencing the delicate balance between these two hormones.

It has been demonstrated that numerous genes that influence various traits including seed color (*MYB10-D* [46]), anthocyanin level (*Dihydroflavonol-4-reductase Gene* (*DFR*) [47]), and dwarfism (*Reduced Height 3* (*Rht3*) [48]), also influence the biosynthesis and transduction of ABA and GA in seeds. This interconnected regulation of ABA and GA by multiple genes has a significant bearing on the development of PHS.

The discovery of PHS genes in vegetable crops is still in its early stages and has largely relied on research conducted in field crops. Only a few genes have been shown to be involved in regulating PHS in vegetables (Table 1). Notably, *VP1*, *VP5*, and *VP10* have been consistently linked to the resilience of cereal crops to PHS. In the context of cucumber, a noteworthy observation is that the expression levels of equivalent genes in prone-to-PHS cucumber varieties are significantly lower compared to those in resistant-to-PHS varieties. Additionally, during different pollination days, the expression levels of *CsVP5* and *CsVP10* were notably lower in materials with easy germination tendencies as opposed to those with difficult germination tendencies. These findings suggest that prone-to-PHS cucumber varieties may suppress the expression of genes related to ABA biosynthesis and signaling pathways, thereby reducing the ABA level and the seeds’ sensitivity to ABA [10]. However, the specific functions of these three genes in cucumber remain to be fully understood. In tomatoes, the downregulation of *METHYLTRANSFERASE 1* (*SlMET1*) causes PHS [6]. It affects ABA content by influencing ABA synthesis and metabolism, ultimately influencing PHS. When combined with numerous physiological experiments in vegetables, it is highly likely that plant hormones governing seed germination play a significant role in regulating PHS in cucumber and other vegetable seeds.

Previous studies have consistently demonstrated the close association between PHS and plant hormones, which are key regulators of seed germination [49,50]. In food crops, identified genes related to PHS are predominantly concentrated in plant hormone pathways. In contrast, research on PHS in vegetable crops is currently focused on gene mapping. Relevant physiological studies and functional annotations of candidate genes within specific QTL regions suggest that plant hormones also play a significant role in influencing the PHS in vegetable crops. The genes that have been verified to function in PHS resistance in cereal crops, such as *VPs*, *ABI3*, *ABI5*, and others, are likely to serve similar roles in vegetable crops. This insight not only highlights the potential universality of these genes in PHS regulation but also offers valuable guidance for the study of PHS in vegetables. As the study of PHS in vegetables progresses, a more comprehensive understanding of the intricate interplay between genetics and plant hormones in regulating PHS is likely to emerge.

### 3.2. Physiological Factors Influencing Pre-Harvest Sprouting in Vegetable Crops through Plant Hormones

PHS in vegetable plants can be influenced by various physiological factors that indirectly affect PHS through their impact on plant hormones. These plant physiological correlates encompass a range of characteristics such as seed coat color, cuticle wax, cell structure, fruit juice osmotic pressure, and plant developmental stage.

In cereal crops, the surface villus and seed-coat cell structure have a substantial impact on seed germination. For instance, in pea beans, prone-to- PHS seeds with an elongated parenchyma structure and smaller stitch length but greater opening degree promote rapid water absorption and faster germination [16]. Similar observations have been made in rice seeds [16,51]. However, it is worth noting that these distinctions may not be present in zucchini, suggesting that seed-coat variations might not significantly impact PHS in zucchini [11]. Plant epidermal wax, with its excellent water retention capacity [52], can also play a role in PHS. In wheat, a segment of a QTL associated with morphological markers linked to PHS resistance has been identified. Notably, the 2B and 2D loci regulate the synthesis of cuticle and wax [53]. Furthermore, it has been observed that the seed dormancy period is shortened and the PHS rate is higher when the color grade of the seed coat is lower [54]. In pea beans, the PHS rate is notably higher in white-grain varieties compared to colored varieties [16], potentially due to variations in flavonoid and carotenoid content in the seed coat. Higher flavonoid levels in the seed coat are inversely correlated with the PHS rate, resulting in a darker seed-coat color [18]. Similarly, higher carotenoid content leads to increased ABA concentration, darker seed-coat color, and reduced PHS rate [55].

The developmental stage of the plant also exerts a significant influence on PHS. In zucchini, PHS is only observed once the seed reaches physiological maturity. It has been established that seeds with higher maturity exhibit lower dormancy and a greater likelihood of germination [11]. Additionally, the developmental stage of the plant itself can affect PHS. For instance, in cucumber, seeds in the fruit are more likely to sprout on wilted or dead plants. Furthermore, late ripening appears to encourage PHS to differing extents. This may be due to inhibitory compounds’, including ABA, being unable to reach the seeds in sufficient quantities, leading to PHS [1,8].

Unlike food crops, most vegetable crop seeds are exposed to a water-rich environment, indicating that the variation in seed-coat structure is not the primary cause of PHS when seeds are surrounded by water. Instead, it has been discovered that the most likely primary factor limiting PHS in such vegetable crops is the osmotic pressure of the fruit juice inside the fruit. The osmotic environment created by the fruit tissue where the seeds are located serves as a barrier to germination [56]. An interesting observation in tomato is that, although there is no significant change in the levels of ABA and GA, the fruit juice of the *rin* mutant has a lower osmolality compared to that of the wild type. This difference in osmotic pressure within the fruit may provide a favorable environment for PHS in the mutant [14]. ABA-deficient mutants exhibit a dramatic reduction in the amount of ABA in seeds, leading to a lower osmotic potential, which is likely a key factor contributing to PHS [13].

In brief, apart from the structure of seed-coat cells and waxes that affect water entry into seeds, plant physiological correlates are also highly likely to affect the transfer of endogenous plant hormones to seeds, thereby affecting PHS. Due to the differences in the surrounding environment of the seeds in some vegetables, people have also put forward a relatively novel view that osmotic pressure can also affect PHS. This innovative concept opens up new avenues for future research [56].

### 3.3. Environmental Factors Affect Pre-Harvest Sprouting by Affecting the Contents of Plant Hormones

PHS is a highly intricate physiological process that can be significantly influenced by various external factors, such as temperature, light, and moisture. These factors not only impact the degree of PHS but also determine when PHS initiates. PHS is sometimes referred to as a climatic calamity, due to its ability to sense and respond to external weather conditions [57].

Temperature has a substantial effect on PHS. For instance, in pea beans, the high-temperature environment under open cultivation led to a higher rate of PHS compared to cultivation under protective measures [16]. During the later stages of cucumber development, the ambient temperature gradually increases. Later pollination leads to higher seed maturity and higher germination rates [1,8]. This suggests that seeds can germinate more quickly at higher temperatures. Similarly, in oilseed rape, PHS rates increase with rising temperatures [58]. Both the synthesis of endogenous phytohormones and the activity of enzymes involved in seed germination are influenced by temperature. For example, low-temperature treatment (4 °C) in rape may inhibit the synthesis of salicylic acid (SA) and the SA signaling pathway [59]. In other crops, temperature affects the expression of genes such as *Delay of Germination1* (*DOG1*) [60], *GA2-Oxidase2*(*GA2ox2*) [61], *GA2-Oxidase5* (*GA2ox5*) [61], *9 cis-Epoxycarotenoid Dioxygenase 1* (*NCED1*) [39], *9 cis-Epoxycarotenoid Dioxygenase 2* (*NCED2*) [61], *Allene Oxide Synthase* (*AOS*) [39], and *Allene Oxide Cyclase* (*AOC*) [39]. This, in turn, impacts the levels of ABA, GA, and jasmonic acid (JA) in the plant, ultimately affecting seed germination.

Water plays a significant role in influencing PHS as it is essential for seed germination. Some researchers suggest that PHS can be triggered by excessive moisture in humid environments where water is transferred to the seeds, resulting in seed germination [62,63,64]. However, in vegetable crops such as cucumber and tomato, where the fruit contains a high water content, it has been hypothesized that the fruits’ own osmotic pressure inhibits germination. Nonetheless, PHS can occur when the fruit begins to rot and takes in water, possibly due to changes in osmotic pressure and other conditions as the fruit deteriorates [1]. The germination rate of rape and kidney bean can be significantly impacted by ambient water levels. Increasing the water content in the cultivation environment can substantially raise the PHS rate in kidney beans and rape [16,58]. In food crops, water also enhances the GA:ABA ratio by effecting the expression of *ABA8oxs, NCED, Late Embryogenesis Abundant 3* (*LEA3*), *GA20-oxidases* (*GA20oxs*), and *GA3-oxidase 2* (*GA3ox2*) genes within the plant, thereby promoting germination [59,65,66].

Seeds have the capability to sense light, which, in turn, influences their dormancy and germination. Arabidopsis seeds promote germination by phytochrome in the presence of red or far-red light [67]. Under blue light, photoreceptor cryptochromes in seeds control ABA synthesis, ABA breakdown, and germination by upregulating 9-cis-epoxycarotenoid dioxygenase and downregulating ABA 8’-hydroxylase [67]. Green light, on the other hand, can prevent the germination of barley seeds through cryptochromes [68]. For more details, refer to the comprehensive review by the authors of reference [69].

In summary, the environment has a multifaceted impact on PHS in plants. It not only influences enzyme activities within plants but also regulates the content and ratio of internal phytohormones, which, in turn, affect PHS. This underscores the significance of plant endogenous hormones as a key factor in controlling PHS. Additionally, in vegetable crops, the degree of PHS may also be influenced by the environment’s effects on plant hormones.

## 4. Conclusions and Future Perspectives

PHS has long been a challenge due to the intricate nature of dormancy physiology. Nevertheless, the majority of investigations have centered on the pivotal role played by two key plant endogenous hormones, ABA and GA, in regulating sprouting. In vegetables crops, PHS research is relatively nascent, with a limited number of studies having been conducted. However, in recent years, with the advancements in breeding technology and an increasing awareness of the value of seeds, research on PHS in vegetables has gained momentum. Various research institutions have undertaken studies, particularly focusing on environmental management and the gene localization of PHS. The findings from these studies highlight that, in horticultural crops like cucumber, the differential genes between those prone to PHS and those resistant to it are significantly enriched in the plant hormone pathway, including auxin, ABA, and ethylene. Notably, among several vegetable crops, there is a substantial difference in the ABA:GA ratio between those susceptible to PHS and those with resistance. These observations underscore the continued significance of ABA and GA as the main factors affecting germination in vegetables. Therefore, future research endeavors in this field can draw inspiration from the progress made in food crops, providing a promising avenue for further exploration and understanding of PHS [8,10].

Since most vegetable seeds are enveloped in a water-rich environment throughout their development, research in the realm of vegetables places a greater emphasis on osmotic potential. This emphasis diverges from the focus in numerous studies on food crops. In this context, the water-soluble plant hormone ethylene, synthesized from *S*-adenosyl-methionine, is of particular interest. Ethylene, while not a direct regulator of seed germination, exerts control over the ABA signaling pathway, thereby indirectly controlling seed germination [70]. It is worth noting that various materials have revealed, through transcriptome and genetic methods, the potential association of ethylene-related genes with PHS [8,20]. In wheat and Arabidopsis, ETR1 promoted both ethylene insensitivity and seed dormancy [71]. Additionally, the latest study in wheat indicates that the ethylene content in prone-to-PHS varieties is significantly higher than that in resistant-to-PHS varieties [49]. These discoveries suggest that ethylene likely plays a role in the regulation of PHS, either directly or indirectly. Therefore, future investigations into genes regulating PHS in vegetables can place greater focus on water-soluble plant hormones like ethylene.

Numerous genes have been identified and their respective functions explored in food crops. However, the depth of research on vegetable crops remains comparatively limited. To address this gap in our understanding and to uncover new genes while comprehending the intricate regulatory networks, multi-omics analysis of the metabolome and transcriptome has emerged as a powerful and promising technique. This approach paves the way for future advancements in breeding and practical applications in vegetable crop production. With a vision toward satisfying market demands by developing novel germplasms, future research endeavors should be directed toward identifying the genes associated with PHS resistance and delving into the intricate regulatory mechanism underpinning PHS resistance in vegetable crops.

## Figures and Tables

**Table 1:** Genes that control Pre-harvest sprouting in vegetable crops have been identified in recent years.

Species	Candidate Gene with Physical Position (bp)/Regulatory Gene	Function	Reference
Chinese cabbage	QTL (Chr3 9,859,000–10,632,000)	-	He, 2021 [18]
Cucumber	QTL (Chr4 19,973,692–20,505,510)	-	Cao et al., 2021 [20]
Oilseed rape	QTLs (*qPHS-2-a*, *qPHS-2-b*, *qPHS-2-c*, *qPHS-2-d*, *qPHS-7*)	-	Feng, 2009 [12]
Tomato	*SlRIN* (NC_015442.3)	Keep osmolality in fruit	Wang, 2016 [14]
Tomato	*SlMET1*(NC_015448.3)	Influences the expression of ABA-related genes	Yao et al., 2020 [6]

## Data Availability

The authors responsible for the distribution of the materials integral to the findings presented in this article are Huazhong Ren (renhuazhong@cau.edu.cn) and Xingwang Liu (liuxw01@cau.edu.cn).

## References

[1] Pang J., Ma D., Huo Z., Li S. Effects of Growth Time and Afterrepining on Maturation and Germination-in-fruit of Cucumber Seeds. Proceedings of the 4th Youth Symposium of Chinese Horticultural Society.

[2] Zhang C., Zhou L., Lu Y., Yang Y., Feng L., Hao W., Li Q., Fan X., Zhao D., Liu Q. (2020). Changes in the physicochemical properties and starch structures of rice grains upon pre-harvest sprouting. Carbohydr. Polym..

[3] Ding Y., Feng F., Xu X., Chen W., Zhou H., Ye C. (2021). The Effect of Vivipary on Fruit Quality of Jackfruit. Southeast Hortic..

[4] Xu F., Tang J., Wang S., Cheng X., Wang H., Ou S., Gao S., Li B., Qian Y., Gao C. (2022). Antagonistic control of seed dormancy in rice by two bHLH transcription factors. Nat. Genet..

[5] Huang T., Qu B., Li H., Zuo D., Zhao Z., Liao Y. (2012). A maize viviparous 1 gene increases seed dormancy and preharvest sprouting tolerance in transgenic wheat. J. Cereal Sci..

[6] Yao M., Chen W., Kong J., Zhang X., Shi N., Zhong S., Ma P., Gallusci P., Jackson S., Liu Y. (2020). METHYLTRANSFERASE1 and Ripening Modulate Vivipary during Tomato Fruit Development. Plant Physiol..

[7] Pang J., Li H., Ma D. (2002). Progress on Preharvest Sproution of Vegetable Crops. Tianjin Agric. Sci..

[8] Tian X. (2021). Evaluation and Transcriptome Analysis of Seed Germination in Melon of Cucumber Germplasm Resources. Ph.D. Thesis.

[9] Guan W., Cao M., Li S., Wang H., Yang R., Deng Q. (2016). Preliminary study on inhibition of preharvest cucumber seed germination by exogenous ABA. Bull. Agric. Sci. Technol..

[10] Mo Y., Wang L., Zhou Y., Sun Q., He W., Chen H., Xiao L., Wang R. (2020). Physiological characteristics and related gene expression of vivipary in cucumber seeds. J. South. Agric..

[11] Li C. (2017). Study on the Vivipary Chracteristics of Summer Squash Seed. Ph.D. Thesis.

[12] Feng F. (2009). Genetic Studies and QTL Mapping of Seed Vivipary in *Brassica napus* L.. Ph.D. Thesis.

[13] Groot S.C.M. (1992). Karssen Dormancy and Germination of Abscisic Acid-Deficient Tomato Seeds—Studies with the Sitiens Mutant. Plant Physiol..

[14] Wang X. (2016). Physiological Mechanism of Vivipary in One Rin Mutant Tomato Accession and Role of Rin in Vivipary. Ph.D. Thesis.

[15] Sun G., Zhang X., Xiao S. (2005). Maternal Effect on Sensitivity of Embryo to ABA and Grain Dormancy in Wheat. J. Triticeae Crops.

[16] Zhang L. (2007). Study on the Seed Vivipary Eharacteris tics of Eomm on Beall (*Pllaseolu svulgaris* L.). Ph.D. Thesis.

[17] Jin Y., Zhu H., Zhang M., Hui M. (2016). Preliminary Studies on Occurrence Time, Position and nheritance Genetics of Seed Vivipary in Chinese Cabbage (*Brassica rapa*). North Hortic..

[18] He X. (2021). Mapping and Mining of Major Genes for Seed Vivipary Trait in Chinese Cabbage. Ph.D. Thesis.

[19] Cao M., Wang H., Deng Q., Yang R., Li S. (2018). Quantitative Genetic Analysis of Pre-harvest Sprouting in Cucumber. Chian Veg..

[20] Cao M., Li S., Deng Q., Wang H., Yang R. (2021). Identification of a major-effect QTL associated with pre-harvest sprouting in cucumber (*Cucumis sativus* L.) using the QTL-seq method. BMC Genom..

[21] Penfield S. (2017). Seed dormancy and germination. Curr. Biol..

[22] Hable W.E., Oishi K.K., Schumaker K.S. (1998). Viviparous-5 encodes phytoene desaturase, an enzyme essential for abscisic acid (ABA) accumulation and seed development in maize. Mol. Gen. Genet..

[23] Singh M., Lewis P.E., Hardeman K., Bai L., Rose J.K.C., Mazourek M., Chomet P., Brutnell T.P. (2003). Activator mutagenesis of the pink scutellum1/viviparous7 locus of maize. Plant Cell.

[24] Maluf M.P., Saab I.N., Wurtzel E.T., Sachs M.M. (1997). The viviparous12 maize mutant is deficient in abscisic acid, carotenoids, and chlorophyll synthesis. J. Experin. Botan..

[25] Bittner F., Oreb M., Mendel R.R. (2001). ABA3 is a molybdenum cofactor sulfurase required for activation of aldehyde oxidase and xanthine dehydrogenase in Arabidopsis thaliana. J. Biol. Chem..

[26] Xiong L.M., Ishitani M., Lee H., Zhu J.K. (2001). The Arabidopsis LOS5/ABA3 locus encodes a molybdenum cofactor sulfurase and modulates cold stress-responsive and osmotic stress-responsive gene expression. Plant Cell.

[27] Leydecker M.T., Moureanx T., Kraepiel Y., Schnorr K., Caboche M. (1995). Molybdenum Cofactor Mutants, Specifically Impaired in Xanthine Dehydrogenase-Activity and Absisic-Acid Biosynthesis, Simultaneously Overexpress Nitrate Reductase. Plant Physiol..

[28] Sagi M., Scazzocchio C., Fluhr R. (2002). The absence of molybdenum cofactor sulfuration is the primary cause of the flacca phenotype in tomato plants. Plant J..

[29] Tan B.C., Schwartz S.H., Zeevaart J.A.D., McCarty D.R. (1997). Genetic control of abscisic acid biosynthesis in maize. Proc. Natl. Acad. Sci. USA.

[30] Suzuki M., Latshaw S., Sato Y., Settles A.M., Koch K.E., Hannah L.C., Kojima M., Sakakibara H., McCarty D.R. (2008). The maize Viviparous8 locus, encoding a putative ALTERED MERISTEM PROGRAM1-like peptidase, regulates abscisic acid accumulation and coordinates embryo and endosperm development. Plant Physiol..

[31] Mccarty D.R., Hattori T., Carson C.B., Vasil V., Lazar M., Vasil I.K. (1991). The VIVIPAROUS-1 Developmental Gene of Mazie Encodes a Novel Transcriptional Activator. Cell.

[32] Hoecker U., Vasil I.K., Mccarty D.R. (1995). Integrated control of seed maturation and germinationprograms by activator and repressor functions of Viviparous-1 of maize. Genes Dev..

[33] Mccarty D.R., Carson C.B., Stinard P.S., Robertson D.S. (1989). Molecular Analysis of viviparous-1: An Abscisic Acid-Insensitive Mutant of Maize. Plant Cell.

[34] Wang R., Chen Y., Sun M., Zhang X., Du Y., Zheng J. (2019). Genetic analysis and causal gene identification of maize viviparous mutant vp-like8. Acta Agron. Sin..

[35] Chu L. (2021). Effect and Mechanism of AP2/ERF Teanscription Factor OsERF44 on Panicle Sprouting in Rice. Ph.D. Thesis.

[36] Bentsink L., Jowett J., Hanhart C.J., Koornneef M. (2006). Cloning of DOG1, a quantitative trait locus controlling seed dormancy in Arabidopsis. Proc. Natl. Acad. Sci. USA.

[37] Sugimoto K., Takeuchi Y., Ebana K., Miyao A., Hirochika H., Hara N., Ishiyama K., Kobayashi M., Ban Y., Hattori T. (2010). Molecular cloning of Sdr4, a regulator involved in seed dormancy and domestication of rice. Proc. Natl. Acad. Sci. USA.

[38] Du L., Xu F., Fang J., Gao S., Tang J., Fang S., Wang H., Tong H., Zhang F., Chu J. (2018). Endosperm sugar accumulation caused by mutation of PHS8/ISA1 leads to pre-harvest sprouting in rice. Plant J..

[39] Xu F., Tang J., Gao S., Chang X., Du L., Chu C. (2019). Control of rice pre-harvest sprouting by glutaredoxin-mediated abscisic acid signaling. Plant J..

[40] Xi W., Liu C., Hou X., Yu H. (2010). Motherof FT and TFL1 Regulates Seed Germination through a Negative Feedback Loop Modulating ABA Signaling in Arabidopsis. Plant Cell.

[41] Zhang L., Wang B., Zang Q. (2007). Progress in Seed Vivipary Mechanism. Chin. J. Cell Biol..

[42] Dai C., Xue H. (2010). Rice early flowering1, a CKI, phosphorylates DELLA protein SLR1 to negatively regulate gibberellin signalling. EMBO J..

[43] Shu K., Zhang H., Wang S., Chen M., Wu Y., Tang S., Liu C., Feng Y., Cao X., Xie Q. (2013). ABI4 Regulates Primary Seed Dormancy by Regulating the Biogenesis of Abscisic Acid and Gibberellins in Arabidopsis. PLoS Genet..

[44] Dill A., Thomas S.G., Hu J.H., Steber C.M., Sun T.P. (2004). The Arabidopsis F-box protein SLEEPY1 targets gibberellin signaling repressors for gibberellin-induced degradation. Plant Cell.

[45] Jacobsen S.E., Binkowski K.A., Olszewski N.E. (1996). SPINDLY, a tetratricopeptide repeat protein involved in gibberellin signal transduction Arabidopsis. Proc. Natl. Acad. Sci. USA.

[46] Lang J., Fu Y., Zhou Y., Cheng M., Deng M., Li M., Zhu T., Yang J., Guo X., Gui L. (2021). Myb10-D confers PHS-3D resistance to pre-harvest sprouting by regulating NCED in ABA biosynthesis pathway of wheat. New Phytol..

[47] Bi H.H., Sun Y.W., Xiao Y.G., Xia L.Q. (2014). Characterization of DFR allelic variations and their associations with pre-harvest sprouting resistance in a set of red-grained Chinese wheat germplasm. Euphytica.

[48] Masaharu S., Mark S.A., Chi-Wah T. (2010). The maize viviparous15 locus encodes the molybdopterin synthase small submit. Pant J..

[49] Zhou G., Wu S., Chen D., Wu X., Cai Q. (2023). Polyphenols and phytohormones profiling of pre-harvest sprouting resistant and susceptible wheat genotypes. SN Appl. Sci..

[50] Sohn S.-I., Pandian S., Kumar T.S., Zoclanclounon Y.A.B., Muthuramalingam P., Shilpha J., Satish L., Ramesh M. (2021). Seed Dormancy and Pre-Harvest Sprouting in Rice—An Updated Overview. Int. J. Mol. Sci..

[51] Cai J.X., Chen W. (2008). Study on the physiological biochemistry of pre-harvest sprouting and scanning electron microscopy of glume in rice. Chin. Agric. Sci. Bull..

[52] Xu J. (2014). Analysis the Cuticular Wax and Influences of Tissue Culture Condition on Cuticular Wax and Water Saving of Tobacco Leaf. Ph.D. Thesis.

[53] Flintham J., Adlam R., Bassoi M., Holdsworth M., Gale M. (2002). Mapping genes for resistance to sprouting damage in wheat. Euphytica.

[54] Shen Z., Yu S., Wu S. (1991). Study on Pre-Harvest Sprouting Resistance in Wheat Cultivars. Sci. Agric. Sin..

[55] Zeevaart J.A.D., Creelman R.A. (1988). Metabolism and Physiology of Abscisic-Acid. Annu. Rev. Plant Physiol. Plant Mol. Biol..

[56] Welbaum G.E., Bradford K.J. (1990). Water Relations of Seed Development and Germination in Muskmelon (*Cucumis melo* L.) V. Water Relations of Imbibition and Germination. Plant Physiol..

[57] He Z., Chen X., Han Y. (2000). Progress on Preharvest Sprouting Resistance in White Wheat. J. Triticeae Crops.

[58] Liu X., Yang Q., Zhang H.Q., He J.W., Yan Y.T., Ju H. (2022). Influencing factors of the vivipary occurrence of rapeseed. J. Hunan Agric. Univ. Nat. Sci..

[59] Liu Z., Zou Y., Dong X., Wei J., Xu C., Mi W., Xu M., Fang X., Cao X., Zheng G. (2021). Germinating seed can sense low temperature for the floral transition and vernalization of winter rapeseed (*Brassica rapa*). Plant Sci..

[60] Nee G., Xiang Y., Soppe W.J.J. (2017). The release of dormancy, a wake-up call for seeds to germinate. Curr. Opin. Plant Biol..

[61] Wang Y., Cui Y., Hu G., Wang X., Chen H., Shi Q., Xiang J., Zhang Y., Zhu D., Zhang Y. (2018). Reduced bioactive gibberellin content in rice seeds under low temperature leads to decreased sugar consumption and low seed germination rates. Plant Physiol. Biochem..

[62] Obroucheva N.V., Sinkevich I.A., Lityagina S.V., Novikova G.V. (2017). Water relations in germinating seeds. Russ. J. Plant Physiol..

[63] Chen B., Liu J. (2017). Research Progress of Rie Vivipary and Its Regulation. Seed.

[64] Sussex I.M. (1975). Growth and metabolism of the embryo and attached seeding of the viviparous mangrove Rhizophora mangle. Am. J. Bot..

[65] Dong Y., Xu H., Zhang H., Zhang H., Wang F., Gu N., Zhu Y. (2022). Dynamic profile of genes related to seed dormancy under high humidity condition during late stage of rice grain filling. J. Zhejiang Agric. Univ..

[66] Toh S., Imamura A., Watanabe A., Nakabayashi K., Okamoto M., Jikumaru Y., Hanada A., Aso Y., Ishiyama K., Tamura N. (2008). High temperature-induced abscisic acid biosynthesis and its role in the inhibition of gibberellin action in Arabidopsis seeds. Plant Physiol..

[67] Barrero J.M., Downie A.B., Xu Q., Gubler F. (2014). A Role for Barley CRYPTOCHROME1 in Light Regulation of Grain Dormancy and Germination. Plant Cell.

[68] Hai H., Sechet J., Bailly C., Leymarie J., Corbineau F. (2014). Inhibition of germination of dormant barley (*Hordeum vulgare* L.) grains by blue light as related to oxygen and hormonal regulation. Plant Cell Environ..

[69] Yang L., Liu S., Lin R. (2019). Advances in Light and Hormones in Regulating Seed Dormancy and Germination. Chin. Bull. Bot..

[70] Ghassemian M., Nambara E., Cutler S., Kawaide H., Kamiya Y., McCourt P. (2000). Regulation of abscisic acid signaling by the ethylene response pathway in arabidopsis. Plant Cell.

[71] Wei J., Wu X., Li X., Wim J.J.S., Cao H., Liu Y. (2023). Overexpression of Taetr1-1 promotes enhanced seed dormancy and ethylene insensitivity in wheat. Planta.

